# GPER1 (GPR30) knockout mice display reduced anxiety and altered stress response in a sex and paradigm dependent manner

**DOI:** 10.1016/j.yhbeh.2014.09.001

**Published:** 2014-09

**Authors:** Iris Kastenberger, Christoph Schwarzer

**Affiliations:** Department of Pharmacology, Innsbruck Medical University, Peter-Mayr-Str. 1a, A-6020 Innsbruck, Austria

**Keywords:** G-protein coupled estrogen receptor, Emotional control, Anxiety, Stress coping, Estrous cycle, Corticosterone

## Abstract

The putative estrogen receptor GPER1 (the former orphan receptor GPR30) is discussed to be involved in emotional and cognitive functions and stress control. We recently described the induction of anxiety-like effects by the GPER1 agonist G-1 upon systemic injection into mice. To contribute to a better understanding of the role of GPER1 in anxiety and stress, we investigated germ-line GPER1 deficient mice.

Our experiments revealed marked differences between the sexes. A mild but consistent phenotype of increased exploratory drive was observed in the home cage, the elevated plus maze and the light–dark choice test in male GPER1 KO mice. In contrast, female GPER1-KO mice displayed a less pronounced phenotype in these tests. Estrous-stage dependent mild anxiolytic-like effects were observed solely in the open field test. Notably, we observed a strong shift in acute stress coping behavior in the tail suspension test and basal corticosterone levels in different phases of the estrous cycle in female GPER1-KO mice.

Our data, in line with previous reports, suggest that GPER1 is involved in anxiety and stress control. Surprisingly, its effects appear to be stronger in male than female mice.

## Highlights

• GPER1 deficient mice display an anxiolytic-like phenotype in males
• GPER1 interferes with cycle-dependent stress coping in females
• GPER1 interferes with cycle-dependent corticosterone levels in females

## Introduction

Mood disorders are the most prevalent cause of morbidity and disability worldwide. The prevalence of depression and anxiety disorders is higher in females than in males. Women are more likely to develop generalized anxiety disorders (7% in women, 4% in men), panic disorders (8% in women, 3% in men) and posttraumatic stress disorders (12.5% in women, 6% in men) (for a review see Lebron-Milad and Milad, 2012). These differences manifest after puberty (for a review see Hayward and Sanborn, 2002), indicating the importance of sexual hormones. A close interaction between mood and hormonal variations may occur during the menstrual cycle by virtue of the cyclic modifications in the synthesis, release and circulating concentrations of gonadal steroids. Although the role of estrogen in cycle dependent mood disorders like premenstrual syndrome or postpartum and menopausal depressive disorders is not entirely clear (Payne, 2003, Westberg and Eriksson, 2008), a broad range of interactions with neuronal signaling has been demonstrated (Wittmann et al., 2009). In most humans, high and constant levels of estrogen are described as anxiolytic and “emotionally positive”. In contrast, low and/or fluctuating levels correlate with dysphoric emotional states and increased anxiety.

Besides humans, anxiogenic and dysphoric effects of estrogen (E2) have also been described in mice, especially with fluctuating levels (Morgan and Pfaff, 2001). The dependence of behavior on the estrus cycle has also been described in rodents (for a review see ter Horst et al., 2012).

The best understood E2 signaling pathways are via the nuclear E2 receptors ERα and ERβ, both ligand-activated transcription factors (Heldring et al., 2007). They primarily regulate transcription by binding to estrogen responsive elements after translocation to the nucleus. Nuclear estrogen receptors most probably are responsible for lasting effects of single pulse E2 treatment (Otto et al., 2012). However, some E2 effects reflect rapid changes in neuronal function by processes initiated at the membrane surface (Boulware and Mermelstein, 2005). Such membrane estrogen receptors were considered to be membrane subpopulations of ERα and ERβ (mERα, mERβ) until the identification of G-protein coupled estrogen receptors. Since 2005 it has been suggested that E2 also binds to and signals through GPER1 (the former orphan receptor GPR30) in vitro, leading to classification of GPER1 by the International Union of Pharmacology as a membrane estrogen receptor (Olde and Leeb-Lundberg, 2009). However, the function of GPER1 as an estrogen receptor is still highly controversial (Langer et al., 2010).

The identification of GPER1 has introduced an additional receptor potentially responsible for non-genomic estrogen signaling. GPER1 has been reported to signal via G_s_ proteins, stimulating cAMP production, and through a pertussis toxin-sensitive G-protein, triggering cleavage of membrane-tethered heparin-bound epidermal growth factor, leading to transactivation of EGF receptors, intracellular Ca^2 +^ mobilization, ERK1/2 activation, and Src activation (Olde and Leeb-Lundberg, 2009).

Both male and female rodents express GPER1 in the brain (for a review see Brinton et al., 2008), with high expression in the islands of calleja and the striatum. GPER1 has also been observed in the hypothalamus (Xu et al., 2009), the pituitary (Hazell et al., 2009), the hippocampal formation, the substantia nigra, the PVN, the basolateral amygdala (Tian et al., 2013) and the supraoptic nucleus (Brailoiu et al., 2007). The localization of GPER1 in the amygdala, hypothalamus and pituitary suggests a role in emotional control and regulation of endocrine responses. Moreover, GPER1 expression in cholinergic neurons of the basal forebrain suggests a role in cognitive functions (Hammond and Gibbs, 2011).

Recently, GPER1 was suggested to play roles in E2-mediated effects on mood, as G15 (a GPER1-selective antagonist) attenuated the effects of G1 (GPER1 selective agonist) and E2 in a mouse model of depression (Dennis et al., 2009), and in cognitive functions such as spatial learning, memory and attention (for review see (Hammond and Gibbs, 2011)). We recently identified GPER1 as a potential mediator of anxiogenic E2 effects in both male and female mice by applying pharmacological tools (Kastenberger et al., 2012b).

However, opinions differ as to whether GPER1 mediates anxiogenic or anxiolytic effects and how it may affect stress related responses based on pharmacological experiments. We now address this question in GPER1-KO mice. The main aim of this study was to investigate the functional role of GPER1 on anxiety and stress coping in both male and female mice. Therefore, we compared anxiety and stress coping behaviors of intact male and female WT and GPER1-KO mice in a battery of tests. Female mice were investigated with respect to their estrous cycle stage and also subjected to analysis of corticosterone serum levels.

## Material and methods

### Mice

GPR30-deficient mice were obtained from Bayer Pharma AG (Otto et al., 2009). KO mice were backcrossed to C57BL/6J mice obtained from Charles River (Sulzfeld, Germany). Littermates were used as controls. A total of 50 wild-type and 50 KO mice were investigated in this study. The genotype was determined by PCR analysis of genomic DNA from tail-tip biopsies.

Age matched male and female mice aged three to eight months were tested in all experiments. All procedures involving animals were approved by the Austrian Animal Experimentation Ethics Board and were performed in compliance with the European Convention for the Protection of Vertebrate Animals used for Experimental and Other Scientific Purposes (ETS no. 123). Every effort was taken to minimize the number of animals used.

### Housing conditions

Male and female mice were kept in the same room but in separate, single ventilated cages (type 2 L), in groups of up to five mice per cage. Mice were kept under controlled conditions (temperature 23 °C, relative humidity of approx. 45%) with lights on (50–60 lx) at 6:30 a.m. and lights off at 6:30 p.m. with free access to food and water (tap water). The cages were bedded with wood chips and a plastic tube as a hiding place.

### Physical exam

Mice underwent a general observation to see if the eyes, whiskers, coat and general movement were normal or altered (Karl et al., 2003). Motor activity and circadian rhythm was analyzed for 3 days using infrared detection in their home cages. That was followed by a noise-reaction-test where the mouse should respond to clapping hands with general muscle contraction, eye blinks and retreating away from the source of sound. Normal functioning of the whiskers was confirmed by touching the whiskers with a thin wire. Muscular strength was analyzed by the wire hang test, in which the mouse had to hang on a cage lid, elevated 30 cm above the cage, for at least 2 min.

### Behavioral testing

All mice were acclimatized to the behavioral facility at least over night in the anteroom of the testing facility before each test. Temperature, humidity and light conditions were set as in the animal house with free access to food and water. Tests were performed according recommendations of Eumorphia (http://empress.har.mrc.ac.uk) (Kastenberger et al., 2012a, Kastenberger et al., 2012b). Age matched mice were tested in a fixed testing schedule with intervals of one week between open field, elevated plus maze, light dark test and tail suspension test. The forced swim test was performed after a break of 3 weeks as a final test.

Mice that underwent the physical exam were not included in the tests for anxiety and stress coping behavior. Mice used for breeding or daily investigations of the estrous cycle were not behaviorally tested. For behavioral tests, the estrous stage of the female mice was determined once immediately after each test by analysis of vaginal smear to avoid additional stress to the animals and any influence on their estrous cyclicity and testing results.

Every test was videotaped and evaluated using the TSE (Bad Homburg, Germany) VideoMot 2 System (OF, EPM, LDT), or manually by an experimenter blinded to the genotype and treatment (TST, FST) of each mouse. In between and prior to each test, the equipment was cleaned with 10% ETOH.

### Vaginal smear evaluation

Immediately after each behavioral test, smears were taken from each female mouse by vaginal lavage with 50 μl saline. The smear was analyzed under an axiophot optical microscope (Carl Zeiss AG, Göttingen, Germany) and dried. Diestrus (also called diestrus II) smears were mainly leucocytic, with some single, round, nucleated epithelial cells. Pro-estrus smears consisted of leukocytes and clumps of nucleated epithelial cells. In estrus smears, denucleated cells with jagged edges and cornified, needle like cells were present. Met-estrus (also called diestrus I), as an intermediate state, consisted of denucleated cells, leucocytes and also sometimes round, nucleated cells. Dried smears were fixed for 10 min in methanol and stained for 45 min in diluted GIEMSA solution (Fa. Roth, Karlsruhe, Germany, Art No. T862.1, diluted 1:20 in distilled water), washed with distilled water and cover slipped (EUKITT, O. Kindler GmbH, Freiburg, Germany) for storage and microscopic analysis.

## Open field test (OF)

The OF test was performed on behaviorally testing naive mice, as it was the first test in the testing battery. The test apparatus was a 50 × 50 cm synthetic box. The arena was divided into 3 areas. The border area was 8 cm distance from the wall, the center (20 × 20 cm) covered 16% of the total area, and the area in between was the intermediate zone. Illumination was set to 150 lx in the center of the OF. When tested, each mouse was placed in the middle of the OF and recorded for ten minutes. The time the mouse spent there, the distance traveled and the number of visits to the center of the OF were taken as measures of trait anxiety levels.

## Elevated plus maze (EPM)

The EPM consisted of 4 arms, forming the shape of a plus, elevated 70 cm above the floor. Two opposing arms were closed by black walls, the other two arms were open. All four arms were connected by a neutral field. The dimensions were 30 × 5 cm for the arms, 5 × 5 cm for the neutral field and the framing of the closed arm had a height of 15 cm.

Illumination in the neutral field was set to 180 lx. Each mouse was placed gently on the neutral field facing an open arm and allowed to explore the maze for 5 min. The time spent and distance traveled in the open arm as well as the number of entries into the open arm were taken as measures of trait anxiety levels. Closed arm entries were analyzed for general motor activity.

## Light dark test (LDT)

The LDT was performed in the OF arena, modified with a black box taking up one third of the space. The illumination in the open area was set to 400 lx. The mouse was set in front of the entrance to the black box and was allowed to explore the area for 10 min. Time spent and distance traveled in the lit area, excluding the transition zone (5 × 5 cm in front of the entrance), and number of entries were measured.

## Tail suspension test (TST)

Mice were fixed with adhesive tape on the tip of their tail to a horizontal metallic bar for 6 min. The light was set to 100 lx in the testing chamber. Immobility was defined as no active movement but breathing for at least two seconds and was analyzed from the 2nd to the 6th minute. Mice who managed to climb up their tail or who fell down were excluded from the analysis.

## Forced swim test (FST)

Two glass cylinders with a diameter of 12 cm and a height of 32 cm were filled with 1.7 l of 25 °C warm tap water directly before the testing. The water was changed and the cylinders were cleaned after each trial.

The mice were dropped into the water from about 30 cm to duck their heads. Due to their fast estrous cycling the test was performed in a single session of 15 min. The behavioral measure scored from the video was the duration of immobility during the 2nd and 6th minute (early immobility) and the last 4 min (late immobility). A mouse was judged to be immobile when making only those movements necessary to keep its head above water for a minimum of 2 s (floating). Mice that were not able to swim (keep their head above the water) for 15 min were excluded from the analysis.

## Stress-induced hyperthermia (SIH)

Mice were allowed to acclimatize to the environment overnight. The stress-induced hyperthermia test started with the first rectal temperature measurement (T(1); basal), followed by a second temperature measurement (T(2)) 10–15 min later. The difference DeltaT (= T(2) − T(1)) is the stress-induced hyperthermia (Olivier et al., 2003). Stress-induced hyperthermia was tested by inserting a glycerol lubricated anal temperature probe (Wittmann et al., 2009). Temperature was recorded when the value stabilized (latest after 10 s). The measurement was repeated 30, 60 and 120 min. after the measurement of basal temperature (T1) to analyze the time-course of the response to restraint.

## Serum analysis

For basal corticosterone measurements, animals naïve to testing were killed between 12:00 and 14:00 o'clock under deep CO_2_ anesthesia by decapitation. A vaginal smear was taken immediately after anesthesia to avoid additional stress before sacrifice occurred. Trunk blood was collected into microcentrifuge tubes and kept for one hour at 4 °C. To obtain serum, blood samples were centrifuged at 1500 rpm for 2 min. The sera were kept frozen in new microcentrifuge tubes at − 20 °C until analyzed. RIA for corticosterone (MP Biomedicals, Eschwege, Germany, corticosterone double antibody — ^125^I RIA Kit, No. 120102) was performed. Diluted sera were analyzed in duplicate according to the manufacturers' guidelines.

## Statistics

Unless stated otherwise, all data are presented as mean ± SEM and the number of animals in brackets. To compare the two genotypes the unpaired t-test was performed.

To compare more than 2 groups with a single genotype, 1 way ANOVA with Bonferroni multiple comparison or Dunnett's post-hoc test was performed.

Different groups with different genotypes were analyzed by 2 way ANOVA followed by a Bonferroni multiple comparison test.

A p-value less than 0.05 was considered to be statistically significant.

All data were analyzed as described by graph pad PRISM 5 for Mac OS X, version 5.0a.

## Results

There were no obvious differences in fertility, body weight, sensory function, fur, whisker condition or estrous smear histology observed between WT and GPER1-KO mice. Estrous cycle length measurements revealed no differences between GPER1-KO and WT mice (WT 6.35 ± 1.4 days and GPER1-KO 6.41 ± 1.6 days).

No difference was observed regarding breeding rates. The number of litters, in one year, of GPER1-KO was indistinguishable from WT mice (GPER1-KO 6.7 ± 2.1 pups per litter, N = 14 litters; WT 7.5 ± 3.3 pups per litter, N = 14 litters; t-test p = 0.49).

Basic behavior in the home cage was normal, without excessive running, jumping or cycling. In addition, when performing the wire hang test, both genotypes were able to hold themselves for a minimum of two minutes on the wire without falling or jumping down.

**Fig. 1:**
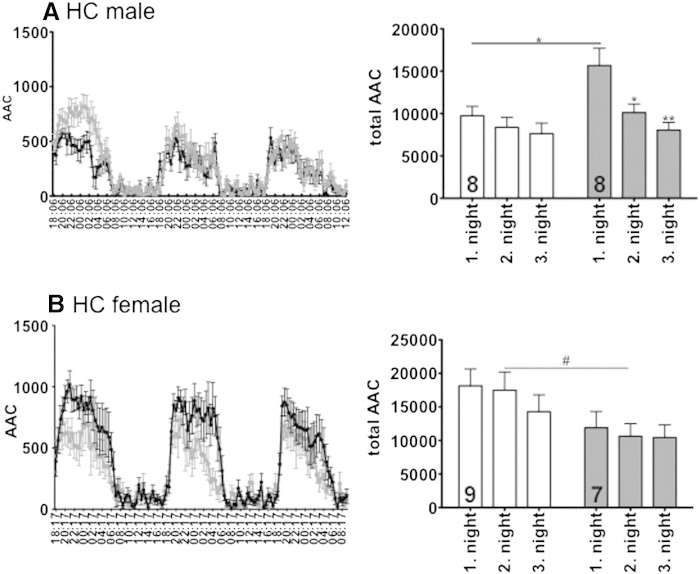
Home cage (HC) activity of male (A) and female (B) WT (black) and GPER1-KO (gray) mice. The left graphs display the circadian rhythmicity of HC activity of male (upper panel) and female (lower panel) WT and GPER1 mice. The bar graphs represent cumulative activities of male and female WT (open bars) and GPER1-KO (shaded bars) mice during three consecutive nights. Data represent mean ± SEM; AAC = arbitrary activity counts. Statistical analysis was done by 1 way ANOVA with Bonferroni post-hoc test: *p < 0.05; **p < 0.01; 2 Way ANOVA with Bonferroni posttest: #p < 0.05. Numbers in bars represent N of animals in testing groups.

In the home cage (HC; Fig. 1), GPER1-KO males showed increased overall activity (2 way ANOVA, F_1,1862_ = 49.15; p < 0.0001), which was mainly due to increased activity during the first night (WT 9737 ± 3143 arbitrary activity counts (AAC), N = 8 vs. GPER1-KO 15,664 ± 5762 AAC, N = 8; unpaired t-test p < 0.0229, 2 way ANOVA p < 0.01, Genotype effect F = 6.452, time F = 7.422; Fig. 1A), which lost statistical significance in the second night (WT 8373 ± 3311 AAC vs. GPER1-KO 10,116 ± 2852 AAC) and was no longer detected by the third night (WT 7626 ± 3509 AAC vs. GPER1-KO 8042 ± 2565 AAC). Ambulation was significantly higher during the first night as compared to the second or third night (1 way ANOVA; F = 5.239; Bonferroni post-hoc test, first vs. second night p < 0.05, first vs. third night p < 0.01) in GPER1-KO, but not WT mice. During daytime there was no difference between the genotypes in activity counts (first day WT 1799 ± 909 AAC vs. GPER1-KO 1611 ± 767 AAC; second day WT 1838 ± 398 AAC vs. GPER1-KO 1935 ± 905 AAC).

In females, WT mice were more active than GPER1-KO mice (F_1,1778_ = 86.70; p < 0.0001). This was observed mainly at night, with a statistically significant difference during the second night (WT 17,488 ± 8148 AAC, N = 9 vs. GPER1-KO 10,635 ± 4971 AAC, N = 7, 2 way ANOVA; F_1,42_ = 7.95; p = 0.0073; Fig. 1B). As in the males, the activity of female GPER1-KO mice was indistinguishable from that of female WT mice during the daytime (WT 2433 ± 1383 AAC, N = 9 vs. GPER1-KO 1560 ± 806 AAC, N = 7, n.s.). Comparing the two sexes, female WT were more active than male WT mice during the night (18,137 ± 7549; 17,488 ± 8148; 14,285 ± 7549 AAC for the first, second and third night of WT (N = 9), respectively vs. 9736 ± 3143; 8372 ± 3311; 7626 ± 3509 for GPER1-KO (N = 7) 2 way ANOVA F_1,45_ = 22.16; p < 0.0001). No such difference was observed for the GPER1-KO mice (F_1,39_ = 0.8448)

During the first 3 h of the HC (novelty), AAC of males and females were comparable (male WT 3639 ± 1044 AAC, N = 8; male GPER1-KO 4331 ± 1354 AAC, N = 8; female WT 4731 ± 2509 AAC, N = 9; female GPER1-KO 3426 ± 2290 AAC, N = 7).

### Anxiety related behavior in male GPER1-KO mice

**Fig. 2:**
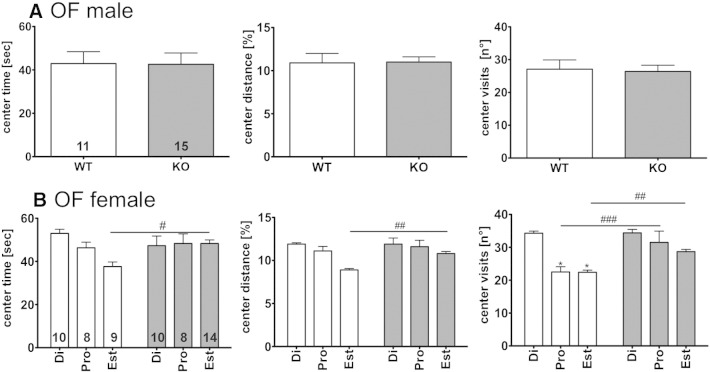
Time spent in the center area (left column), distance traveled in the center (middle column) and entries into the center (right column) during the open field (OF) test are depicted for male (A) and female (B) WT (open bars) and GPER1 KO (shaded bars) mice. Data represent mean ± SEM; 2 way ANOVA with Bonferroni post-hoc test: #p < 0.05; ##p < 0.01; ###p < 0.001. Di = diestrus state; pro = pro-estrus state; est = estrus state. Numbers in bars represent N of animals in testing groups.

In the OF test we did not observe any difference between WT and GPER1-KO mice regarding time spent (WT 43 ± 6 s, N = 11 vs. GPER1-KO 43 ± 5 s, N = 15) or distance traveled (% of total distance WT 11 ± 1% vs. GPER1-KO 11 ± 0.6%) in the center of the OF (Fig. 2A). The numbers of visits to the center were also indistinguishable between the genotypes (WT 27 ± 3 vs. GPER1-KO 26 ± 2).

**Fig. 3:**
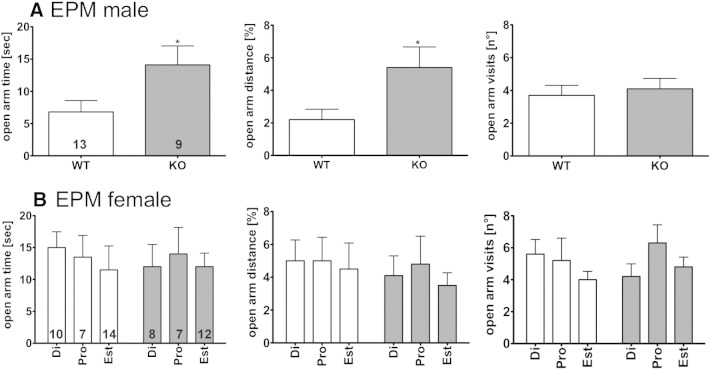
Time spent (left column), distance traveled on the open arm (middle column) and entries into the open arm (right column) during the elevated plus maze (EPM) test are depicted for male (A) and female (B) WT (open bars) and GPER1 KO (shaded bars) mice. Data represent mean ± SEM. Statistical comparisons in A were done by unpaired t-test (*p < 0.05). Di = diestrus state; pro = pro-estrus state; est = estrus state. Numbers in bars represent N of animals in testing groups.

In the EPM (Fig. 3A) we observed increased ambulation of the open arms in the GPER1-KO mice compared with WT mice. This was statistically significant regarding time spent in the open arm (WT 7 ± 2 s, N = 13 vs. GPER1-KO 14 ± 3 s, N = 9, unpaired t-test p = 0.0352,) and the distance traveled (WT 2 ± 1% of total distance vs. GPER1-KO 5 ± 1%, unpaired t-test p = 0.0228). No difference was observed in the number of closed arm entries (WT 14 ± 1 vs. GPER1-KO 15 ± 2), suggesting that overall motor activity was not altered between the genotypes.

**Fig. 4:**
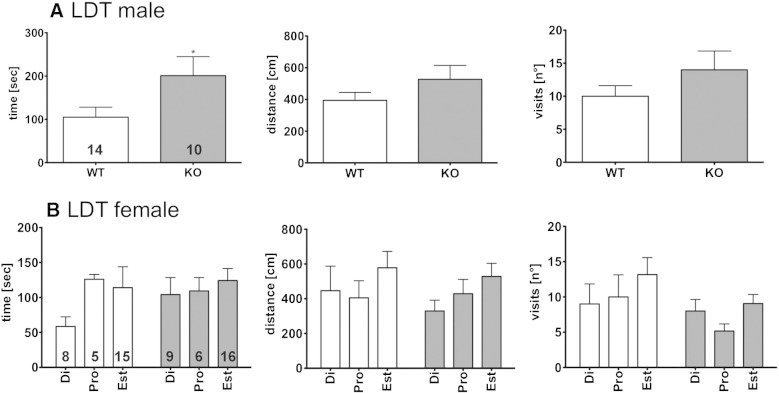
Time spent (left column), distance traveled (middle column) and entries into the lighted area (right column) during the light–dark choice (LD) test are depicted for male (A) and female (B) WT (open bars) and GPER1 KO (shaded bars) mice. Data represent mean ± SEM. Statistical comparisons in A were done by unpaired t-test (*p < 0.05), and in B by applying 2 way ANOVA with Bonferroni post-hoc test # p < 0.05. Di = diestrus state; pro = pro-estrus state; est = estrus state. Numbers in bars represent N of animals in testing groups.

In the LDT (Fig. 4A) we observed a statistically significant difference between WT and GPER1-KO mice in the time spent in the lit area (WT 105 ± 23 s, N = 14 vs. GPER1-KO 201 ± 44 s, N = 10, unpaired t-test p = 0.0481), but not for the distance traveled (WT 394 ± 50 cm vs. GPER1-KO 527 ± 88 cm) or visits to the lit area (WT 10 ± 2 vs. GPER1-KO 14 ± 3).

### Anxiety related behavior in female GPER1-KO mice

Female mice were analyzed as an entire group as well as sub-groups at different estrous stages. Only minor differences were observed between female WT and GPER1-KO mice in the OF. Thus, only during estrus, GPER1-KO mice displayed higher exploratory drive than WT animals in the center area (WT: di 53 ± 6.0 (N = 10); pro 46 ± 7.4 (N = 8); est 38 ± 6.4 (N = 9) s vs. GPER1-KO: di 47 ± 12.6 (N = 8); pro 48 ± 12.6 (N = 8); est 48 ± 6.2 (N = 14) s; 2 way ANOVA with Bonferroni post-hoc test p < 0.05; interaction F_2,51_ = 4.17, estrous stage F_2,51_ = 3.26). However, this was apparently due to reduced exploratory drive in WT mice during this estrous stage, rather than to an increased drive in GPER-KO mice (Fig. 2B). No differences were observed in the EPM (Fig. 3B) and LDT (Fig. 4B).

### Stress related behavior in male GPER1 KO mice

**Fig. 5:**
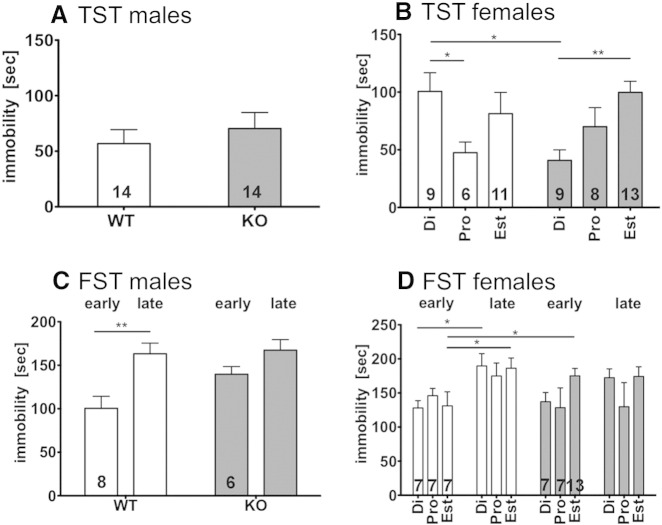
Immobility time (2nd to 6th minute) measured during tail suspension tests (TST) on male (A) and female (B) WT (open bars) and GPER1-KO (shaded bars) mice are shown in the upper panel. Note the inverse results for female mice along the estrous cycle. For the forced swim test (FST) early (2nd to 6th minute) and late (11th to 15th minute) immobility times are displayed for male (C) and female (D) mice. Data represent mean ± SEM; Di = diestrus state; pro = pro-estrus state; est = estrus state; *p < 0.05; **p < 0.01; 2 way ANOVA with Bonferroni post-hoc test. Numbers in bars represent N of animals in testing groups.

In the TST no difference in immobility time was observed between male WT and GPER1-KO mice (WT 57 ± 12 s, N = 14 and GPER1-KO 70 ± 14 s, N = 14, unpaired t-test p = 0.6239; Fig. 5A).

In the FST (Fig. 5C) no difference was observed in the early immobility time (2nd to 6th minute) (GPER1-KO 140 ± 22 s, N = 6 vs. WT 101 ± 39 s, N = 8) or in the last 5 min of the 15-minute trial (GPER1-KO 151 ± 58 s, N = 6 vs. WT 163 ± 34 s, N = 8). However, there was a significant difference between early and late immobility in WT, but not GPER1-KO mice (2-way ANOVA: WT p < 0.01, GPER1-KO p > 0.05; F_1,24_ = 12.98)

**Fig. 6:**
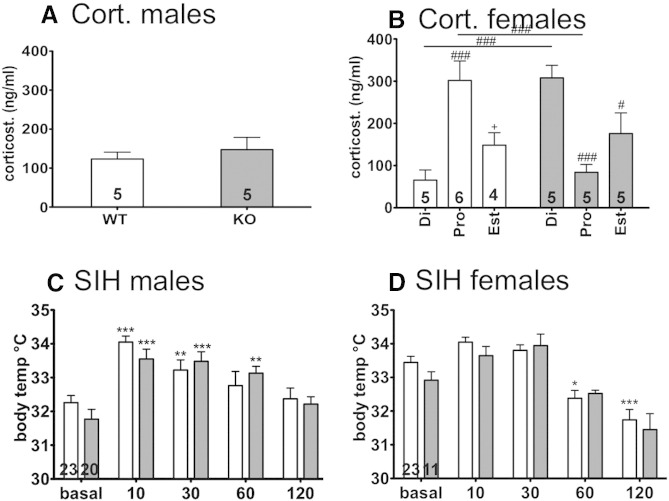
Corticosterone levels in male (A) and female (B) WT (open bars) and GPER1-KO (shaded bars) mice were measured by RIA. Note the antidromic elevation in female WT and KO mice. Stress-induced hyperthermia was measured after 10, 30, 60, and 120 min. Data for male (C) and female (D) WT and GPER1-KO mice are depicted. Note the prolonged increase in stress response in male GPER1-KO mice. Mean ± SEM. Di = diestrus state; pro = pro-estrus state; est = estrus state; mean ± SEM; 2 way ANOVA with Bonferroni post-hoc test #p < 0.05; ##p < 0.01; ###p < 0.001; 1 way ANOVA with Dunnetts's Multiple Comparison Test *p < 0.05. Numbers in bars represent N of animals in testing groups.

Stress-induced hyperthermia revealed comparable basal temperature and initial increases in WT and GPER1-KO mice (Fig. 6C). Noteworthy, the temperature in WT mice had returned to basal after 60 min, while it was still significantly increased in GPER1-KO mice at this time-point. Still, there was no statistical difference between genotypes at any time-interval.

### Stress related behavior in female GPER1 KO mice

In the TST, female WT mice showed a reduced immobility time in pro-estrus compared to diestrus (pro-estrus 48 ± 23 s, N = 6 vs. diestrus 101 ± 49 s, N = 9, 2 way ANOVA p < 0.05; F_2,50_ = 2.407). In contrast, GPER1-KO mice showed reduced immobility time in diestrus (diestrus 41 ± 27 s, N = 9 vs. estrus 100 ± 35, N = 13, 2 way ANOVA;p < 0.01, F_2,50_ = 4.861), resulting in a significant difference between WT and GPER1-KO mice in the diestrus states (2 way ANOVA; Interaction F_2,50_ = 5.02; p < 0.0103).

In the FST no difference regarding estrous state or time was observed in GPER1-KO mice. However, WT mice had a statistically significant increase in immobility time in the last 5 min of the trial compared to the first minutes (WT diestrus early 124 ± 24 s, N = 7 vs. WT diestrus late 190 ± 33 s, N = 7, WT estrus early 124 ± 50 s, N = 7 vs. WT estrus late 183 ± 35 s, N = 7, 2 way ANOVA, F_3,84_ = 6.120, both p < 0.05). This results in an overall significant influence of time (2-way ANOVA, F_3,84_ = 6.120, p = 0.0008). Moreover, we observed a significant difference between WT and GPER1-KO mice in early immobility during estrus (WT estrus early 124 ± 50 s, N = 7 vs. GPER1-KO estrus early 175 ± 39 s, N = 13, 2 way ANOVA, p < 0.05) Three WT mice (two pro-estrus state, one estrous state) were not able to swim for 15 min and were excluded from the analysis.

During the test for stress-induced hyperthermia, we observed higher basal body temperature in female mice as compared to male mice; however, temperatures were comparable between WT and GPER1-KO mice (Fig. 6D). In females, the initial increase was less pronounced than in male mice, but followed by a late decrease in body temperature. The temperature in WT mice was significantly increased 10 min after measuring basal temperature and reduced after 60 min. In contrast, GPER1-KO mice displayed a delayed increase, reaching statistical significance at the 30 min interval, and a delayed subsequent decrease lacking significance at the 60 min interval (Fig. 6D). However, no difference between genotypes was observed at any time-point.

## Serum analysis

Corticosterone levels were measured in male and female GPER1-KO and WT mice. Male mice did not show differences in their basal corticosterone levels (WT 123 ± 17 ng/ml, N = 5 vs. GPER1-KO 147 ± 32 ng/ml, N = 5, Fig. 6A). The basal corticosterone levels in female GPER1-KO mice did not differ from WT mice when all mice were pooled (WT 182 ± 34 ng/ml, N = 15 vs. GPER1-KO 189.3 ± 30.8 ng/ml, N = 15). In contrast, analysis of corticosterone levels according to the estrous cycle (Fig. 6B) showed that WT mice had an increase in corticosterone in pro-estrus (WT diestrus 66 ± 54 ng/ml, N = 5, vs. WT pro-estrus 302 ± 113 ng/ml, N = 5, p < 0.01, 1 way ANOVA; WT pro-estrus 302 ± 113 ng/ml, N = 5 vs. WT estrus 148 ± 59 ng/ml, N = 5, p < 0.05, 1 way ANOVA; F = 11.05; 2 way ANOVA, Interaction F_2,24_ = 19.97 p < 0.0001), whereas GPER1-KO mice showed a decrease in pro-estrus compared to diestrus mice (KO diestrus 308 ± 66 ng/ml, N = 5 vs. KO pro-estrus 84 ± 42 ng/ml, N = 5, p < 0.01, 1 way ANOVA; F = 10.48). There was a significant difference between GPER1-KO diestrus and WT diestrus mice (308 ± 66 ng/ml, N = 5 vs. 66 ± 54 ng/ml, N = 5, 2 way ANOVA, p < 0.001) and GPER1-KO pro-estrus and WT pro-estrus mice (84 ng/ml ± 42, N = 5 vs. 302 ng/ml ± 113, N = 6).

## Discussion

Our data obtained from behavioral testing revealed a phenotype of sex and paradigm dependent reduced anxiety-like behavior and altered stress response in GPER1-KO mice. Alterations in anxiety-like behavior were observed predominantly in male mice, while alterations in stress response were observed in females. The main observation was a shift in the cycle-dependent fluctuations of basal corticosterone levels and stress coping in female GPER1-KO mice. The length of the estrous cycle and duration of the different stages was comparable between GPER1-KO and WT C57BL/6J mice. In any case, cycle lengths were comparable to observations in a longitudinal study of estrous cyclicity in aging C57BL/6J mice (Nelson et al., 1982).

Increased exploratory drive in the GPER1-KO is in line with the anxiogenic effects observed after treatment of male mice with either 17β-est or G1 in our previous study (Kastenberger et al., 2012b). The phenotype of reduced anxiety like behavior was mainly observed in the test with higher aversiveness of the exposed area (EPM and LDT) than in the OF, suggesting that increased stress during the test plays a role. In line with this, WT mice displayed significantly less immobility in the early stage of the FST. High activity in the first minutes is often a sign of anxiety. Still, the effects observed on the C57BL/6N background in the recent pharmacological study (Kastenberger et al., 2012b) were more clear-cut than the effects observed in the germ-line KO mice on the C57BL/6J background used in this study. This may, at least in part, be due to the known differences in anxiety-like behavior between these two strains (Matsuo et al., 2010, Simon et al., 2013). In contrast to C57BL/6N mice, female C57BL/6 J mice used in this study displayed negligible alterations in exploratory behavior along their estrous cycle. Thus, direct implications of altered estrogen levels on alterations in behavior could not be assessed in these animals. In line with alterations in anxiety during the menstrual cycle in human beings (Toufexis et al., 2006), alterations in anxiety-related tests were also reported for female rodents (Frye et al., 2000, Marcondes et al., 2001, Morgan and Pfaff, 2002). While anxiety levels appeared to be lower during late pro-estrus and estrus, indicators of increased anxiety were observed during late diestrus/early pro-estrus, the time of rising estrogen in female mice and rats. It was suggested that these mood changes might be due to the anxiolytic effects of larger amounts of estrogen, which are preceded by the anxiogenic effects of fluctuations in estrogen levels (Toufexis et al., 2006). However, alterations in anxiety and responsiveness to mild anxiogenic stress in diestrus, compared to pro-estrus and estrus, appear highly divergent in both rats and mice (Mora et al., 1996, Ho et al., 2001, Marcondes et al., 2001, Hiroi and Neumaier, 2006). In any case, our present data support the idea that the G1-induced anxiogenic effects in C57BL/6 N mice are indeed GPER1-mediated.

We observed an interesting phenotype regarding the behavior of mice in their home cages. It is known that female mice are more active than male mice, which has been suggested to be related to the higher estrogen levels in females (for a review see (Walf and Frye, 2006)). In fact, ovariectomized female mice displayed strongly reduced home cage activity (Kastenberger et al., 2012b). Sex differences in home cage activity were also observed in this study (55,500 ± 7500 in females vs. 30,700 ± 3500 total activity counts over three nights, two days in males). Noteworthy, GPER1-deficiency induced increased activity in males, but decreased activity in female mice, completely abolishing the difference (39,200 ± 6100 in females vs. 39,400 ± 3200 total activity counts over three nights, two days in males). Thus, considering GPER1 as an estrogen receptor, it could be involved in the locomotive actions of estrogen in females. In contrast, males displayed increased motor activity when they were deficient for GPER1. In addition, there were clear differences between the three subsequent nights of home cage activity testing. The genotype differences were most pronounced during the first night, when the cage was novel. Exploration of a novel environment strongly involves the hippocampus, an area known to be heavily influenced by estrogen (for review see (McEwen et al., 2012)) and to express GPER1 (Hazell et al., 2009, Akama et al., 2013).

Apparently, GPER1 mediates several effects, which sum up to different net-effects in female and male mice. Such differences may depend on higher estrogen levels and resulting differences in activation of different estrogen receptors. It is tempting to speculate that there might be a reduction in anxiety paralleled by a reduction in motor activity in GPER1-KO mice. With low estrogen, higher anxiety and lower motor activity levels – as in males – the sum of the effects reveals an anxiolytic-like phenotype. In contrast, with higher estrogen and high basal motor activity, but lower trait anxiety – i.e. in females – reduced anxiety does not translate into measurable differences under normal conditions. Yet, under basal conditions the reduced motor drive will be the main effect measured. Importantly, no significant differences in total distance traveled were observed between genotypes during behavioral testing.

In the tail suspension test, no differences between genotypes were observed in male mice. The results obtained from female mice in this test revealed marked differences between GPER1-KO and WT mice in the diestrus stage. Differences between the sexes in stress response, with a stronger influence of vasopressin, ACTH and corticosterone on the HPA axis in females, was proposed based on studies in rodents (for a review see ter Horst et al., 2012). Such a difference at low estrogen levels is not supported by the data obtained from the forced swim test, and also not by the data obtained from male mice. Measurement of basal corticosterone levels revealed, that at stages with lowest immobility (pro-estrus in WT and diestrus in GPER1-KO), basal corticosterone is highest. As observed in WT mice, rodents usually display an increase in corticosterone during the pro-estrus and estrus phase (Rodrigues et al., 1995, Atkinson and Waddell, 1997). Also, the high levels of basal serum corticosterone measured were in line with the literature (Rodrigues et al., 1995). GPER1-KO mice contrast this by displaying increased basal corticosterone levels during diestrus and the lowest basal levels during pro-estrus. Noteworthy, pooling the data of all female mice tested without separating the estrous cycle stages results in unaltered stress behavior and corticosterone levels.

The results regarding stress induced hyperthermia revealed minor alterations in the GPER1-KO mice. If there was a difference at all, it was a potentially prolonged stress response observed in stress-induced hyperthermia consistent between the two sexes. The early response (increase after 10 min) is comparable between genotypes, which is in line with the lack of difference observed in male mice in the tail suspension and forced swim tests and in females in the forced swim test. The higher basal temperature and resulting less pronounced stress-induced increase in female mice is in line with the literature (Sanchez-Alavez et al., 2011).

Overall, our data indicate that GPER1 is involved in sex steroid dependent stress and corticosterone-level control, thereby supporting the hypothesis that GPER1 indeed represents an estrogen receptor. However, the mechanism(s) involved appear complex and may well involve counteracting mechanisms in the hypothalamus and hippocampus as well as the spinal cord. Moreover, GPER1 is involved in anxiety-like behavior; however, without displaying any clear estrogen dependency of these effects. Thus, our data provide clear evidence for a functional role of GPER1, but to understand the complex nature of this involvement further experiments are needed. These experiments should focus on the functional role of GPER1 in distinct brain nuclei and therefore depend on the generation of new tools like conditional GPER1 KO mice.
